# Synthesis of novel MoWO_4_ with ZnO nanoflowers on multi-walled carbon nanotubes for counter electrode application in dye-sensitized solar cells

**DOI:** 10.1038/s41598-022-16791-2

**Published:** 2022-07-21

**Authors:** Yonrapach Areerob, Chaowalit Hamontree, Phitchan Sricharoen, Nunticha Limchoowong, Supinya Nijpanich, Theeranuch Nachaithong, Won-Chun Oh, Kongsak Pattarith

**Affiliations:** 1 Department of Industrial Engineering, School of Engineering, King Mongkut’s Institute of Technology Ladkrabang, Bangkok, 10520 Thailand; 2 Department of Premedical Science, Faculty of Medicine, Bangkokthonburi University, Thawi Watthana, Bangkok, 10170 Thailand; 3 Department of Chemistry, Faculty of Science, Srinakharinwirot University, Bangkok, 10110 Thailand; 4 Synchrotron Light Research Institute (Public Organization), 111 University Avenue, Muang District, Nakhon Ratchasima, 30000 Thailand; 5 Department of Physics, Faculty of Science, Khon Kaen University, Khon Kaen, 40002 Thailand; 6 College of Materials Science and Engineering, Anhui University of Science and Technology, Huainan, 232001 People’s Republic of China; 7 Department of Advanced Materials Science and Engineering, Hanseo University, Seosan-si, 31962 Chungcheongnam-do South Korea; 8 Department of Chemistry, Faculty of Science, Buriram Rajabhat University, Buriram, 31000 Thailand

**Keywords:** Materials science, Materials for energy and catalysis, Solar cells

## Abstract

Novel MoWO_4_ with ZnO nanoflowers was synthesized on multi-walled carbon nanotubes (MW-Z@MWCNTs) through a simple hydrothermal method, and this unique structure was applied as a counter electrode (CE) for dye-sensitized solar cells (DSSC) for the first time. The synergetic effect of ZnO nanoflowers and MoWO_4_ on MWCNTs was systematically investigated by different techniques. The amount of MWCNTs was optimized to achieve the best DSSC performance. It was found that the 1.5% MW-Z@MWCNTs composite structure had the highest power conversion efficiency of 9.96%, which is greater than that of traditional Pt CE. Therefore, MW-Z@MWCNTs-based CE can be used to replace traditional Pt-based electrodes in the future.

## Introduction

Electricity shortage is a long-standing problem all over the world due to the growing population every year. It is reported that most of the electric power comes from natural gas fuel (70%) followed by lignite and coal, causing serious environmental pollution problems, such as global warming and acid rain. Moreover, these energy sources will run out in the next 18 years; thus, there is a huge need for renewable energies^1^.

Solar cells are popular alternative energy sources because they are clean and can be used indefinitely. Among different solar cell generations, dye-sensitized solar cells (DSSC) have received considerable attention due to their inexpensive, non-toxicity, and easy manufacturing process^2^. Generally, DSSC is made of a mesoporous titanium dioxide (TiO_2_) thin film with dye molecules (N719), an electrolyte of iodide/triiodide (I^−^/I_3_^−^) couple, and a counter electrode (CE). In DSSC, excited dye molecules inject an electron into the conduction band (CB) of nanocrystalline TiO_2_ under exposure to light, and these oxidized dye molecules are subsequently reduced back to their original neutral state by electron donation from I^−^ ions. The CE transfers electrons flowing from the external circuit to the redox reaction electrolyte and catalyzes the reduction of I_3_^−^ ions^3–5^. However, in DSSC, platinum (Pt) is often used as CE, which is an expensive material and unsuitable for industrial production. In order to overcome this problem, different allotropes of carbon^6^, transition metal oxides^7^, transition metal selenide^8^, conductive polymers, and nanocomposites^9^ are used. A suitable CE should have higher catalytic effectiveness, a narrow bandgap, numerous catalytic active sites, good conductivity, fast electron-transfer channels, and charge transport properties. Unfortunately, research on the improvement of CE materials to increase the power conversion efficiency of DSSC is very limited.

Transition metal oxides nanomaterials such as TeO_2_, Bi_2_O_3_, MoO, WO_4_, BaO, etc., are used for the development of photovoltaic advanced materials, photocatalyst and smart device^9^. Among the metal oxides, tungsten oxide, WO_4_, is n-type semiconductor with a small bandgap of 2.6 eV which has unique thermal, optical, physic-chemical, absorbing ability and electrical proper. WO_4_ can be used as electron and hole transport layer due to its high carrier mobility, which will help to improve the carrier transport performance in the DSSC device. However, the photovoltaic applications of WO_4_ are limited because of its unfavorable conduction band edge position for one-electron reduction of O_2_ and hydrogen reduction reactions. This limitation leads to the fast electron–hole recombination rate and the lower photovoltaic and photocatalytic activity. Therefore, in order to prevent this constraint, the research team was interested in the combination of metal oxides and other materials. Gomathi et al. prepared non-toxic Ni-doped MoO_3_ nanostructures for CE through a facile hydrothermal route and obtained a power conversion efficiency (PCE) of 8.39% due to the high electrocatalytic activity of MoO_3_. Two-dimensional ZnO nanoflowers also have good visible transmittance, high chemical stability, excellent morphological properties, and high electron mobility (115–155 cm^2^)^10^. Rotaba Ansir et al. synthesized Pd@C with ZnO nanorods as a photoanode through a microwave treatment and the co-precipitation method and obtained an efficiency of ~ 3.60% due to the reduction in electron–hole recombination and the increase in the number of photo-generated e^−^/h^+^ ^11^.

In recent years, multi-walled carbon nanotubes (MWCNTs), due to their high surface area, excellent chemical stability, and high electrical conductivity, have been used to improve the properties of metal oxides. In 2021, Mahin Mirzaei and Mohammad Bagher Gholiv synthesized functionalized MWCNTs-encapsulated Ni-doped molybdenum diselenide (f-MWCNTs@NiMoSe_2_) by a hydrothermal route^12^ and found that the DSSC fabricated with f-MWCNTs@NiMoSe_2_ had an excellent catalytic activity with a high efficiency of 7.39%. The high PCE of MWCNTs@NiMoSe_2_ could be attributed to the presence of a large number of active sites and the good electrical conductivity of MoSe_2_. Therefore, in this work, novel MoWO_4_ with ZnO nanoflowers were synthesized on MWCNTs (MW-Z@MWCNTs) by a facile hydrothermal route for CE application in DSSC. The optimum content of MWCNTs was determined to achieve high PCE from DSSC.

## Methods

### Materials and reagents

MWCNTs with ≥ 95% purity (external diameter = 10–20 nm and length = 5–10 µm) were purchased from Sigma-Aldrich. Molybdenum oxide (MoO_3_; 99.97% trace metal basis), tungsten (VI) oxide powder (99.9%), ammonium sulfate ((NH_4_)_2_SO_4_; ACS reagent; ≥ 99.0%), zinc nitrate hydrate (Zn(NO_3_)_2_.6H_2_O; 99.99% trace metal basis), hexamethylenetetramine (HMTA), and hydrochloric acid (HCl) were obtained from Merck. Fluorine-doped SnO_2_ (FTO; 15 Ω) transparent glass and Surlyn thermoplastic polymer were procured from Solaronix. All these compounds were of analytical grade.

### Synthesis of MW-Z@MWCNTs

MoWO_4_ was prepared through a simple hydrothermal route^13^. First, 3.29 g of MoO_3_ and 0.5 g of WO_3_ were dissolved in 70 mL of DI water under vigorous stirring for one hour. Subsequently, HCL (3 M) was slowly dropped in the solution until the value reached about 2. Further, 3.30 g of (NH_4_)_2_SO_4_ was added to the resultant solution, stirred continuously for 30 min before placing into a Teflon-lined autoclave enclosed in a stainless-steel tank, and held at 190 °C for 5 h. Finally, the solution was cooled at room temperature, washed several times with water and ethanol, and dried in the air at 90 °C for 12 h.

In order to ZnO nanoflower powder, 0.7 g of Zn(NO_3_)_2_·6H_2_O was dissolved in 75 mL of DI water, and 0.3 g of HMTA was dropped into the resultant solution. Subsequently, 0.9 g of NaOH was dissolved in 40 mL of DI water and added dropwise into the as-prepared solution. The solution was then continuously stirred at 500 rpm for 1 h at room temperature, kept in a Teflon-lined autoclave at 150 °C for 8 h, and finally, washed twice with water and dried at 80 °C overnight^14^.

**Figure 1:**
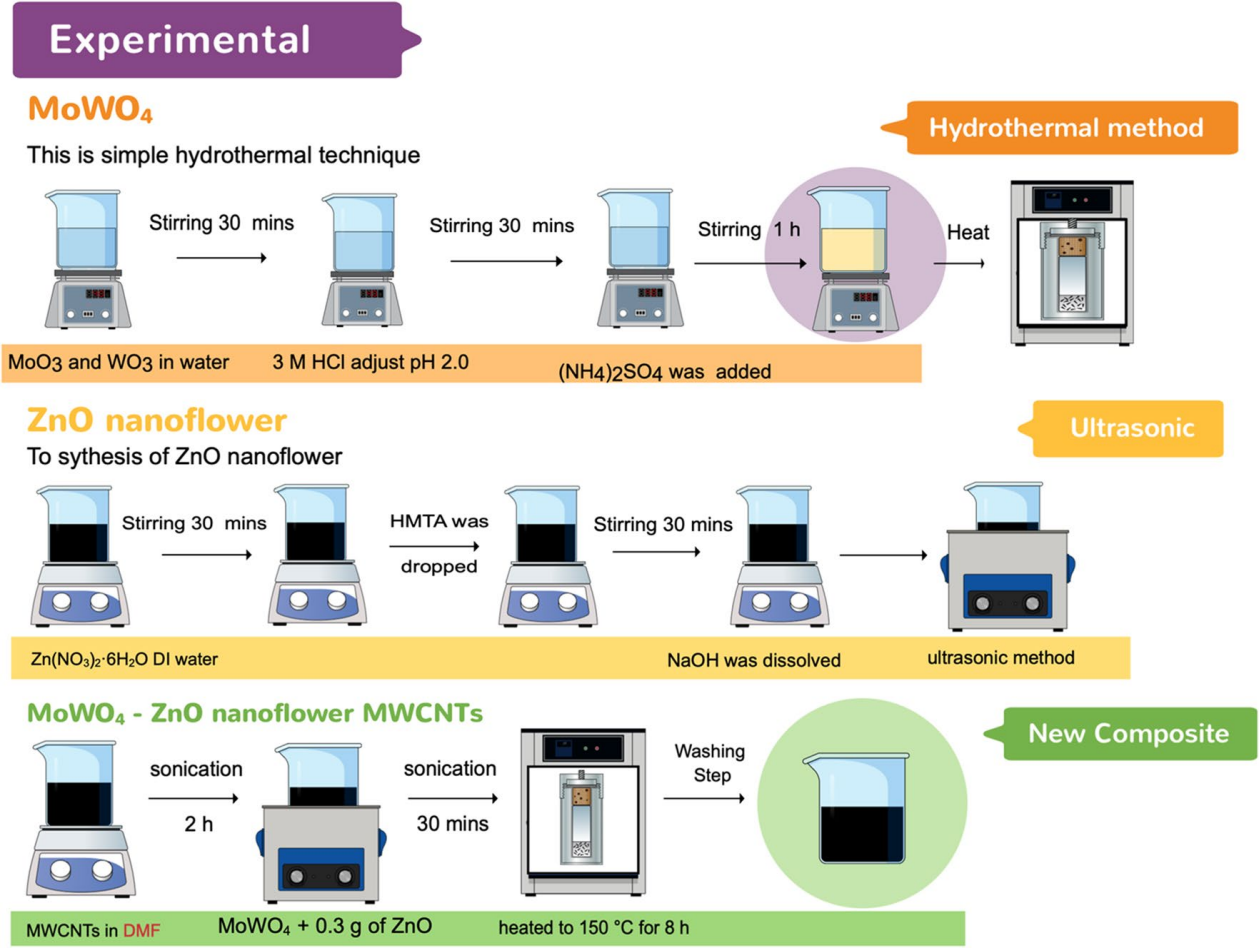
Schematic diagram of MW-Z@MWCNTs synthesis.

In order to synthesize MW-Z@MWCNTs, 0.5 g of MWCNTs was added to 75 mL of dimethylformamide (DMF) under sonication for 2 h. Subsequently, 0.25 g of MoWO_4_ and 0.3 g of ZnO nanoflower powder were added into the mixture solution and continuously sonicated for 30 min. The resultant solution was kept in a Teflon-lined autoclave at 150 °C for 8 h, then washed several times with water and ethanol, and finally, placed in a hot-air oven at 80 °C for 24 h. The schematic diagram for MW-Z@MWCNTs fabrication is displayed in Fig. 1.

### Fabrication of DSSC cell

The Doctor blade method was used to prepare the TiO_2_ paste photoanode and the MW-Z@MWCNTs CE with I^−^/I_3_^−^ pairs of the liquid electrolyte on the FTO substrate. The TiO_2_ paste was soaked in a ruthenium (N719) solution overnight, and the MW-Z@MWCNTs CE was deposited on the FTO substrate by the spin coating technique. A Surlyn film (approximately 30 µm) was used to clip the TiO_2_ paste photoanode and the MW-Z@MWCNTs CE together in a sandwich-type cell.

### Characterization

The phase compositions of the resultant composites were detected by X-ray diffraction analysis (XRD) under Cu-Kα1 radiation at 1.2 kVA (Rigaku, SmartLab). The morphology and surface characteristics of the composite samples were determined by a Fourier-transform scanning electron microscope (FE-SEM; ThermoFisher Scientific, Apreo2, Germany), and their surface elemental composition and oxidation state were analyzed by X-ray photoelectron spectroscopy (XPS; PHI5000 VersaProbe II ULVAC-PHI, Japan) at Synchrotron Light Research Institute (SLRI), Thailand. A monochromatized Al-Kα X-ray source (hγ = 1486.6 eV) was used to excite the samples. Electrochemical impedance spectroscopy (EIS) was conducted in a frequency range from 0.01 to 100 kHz at a bias voltage of 0 V and an amplitude of 10 mV. Current density–voltage (J–V) characteristics were measured by a Keithley 2400 solar simulator under 100 mW/cm^2^ illumination.

## Results

### XRD analysis

**Figure 2:**
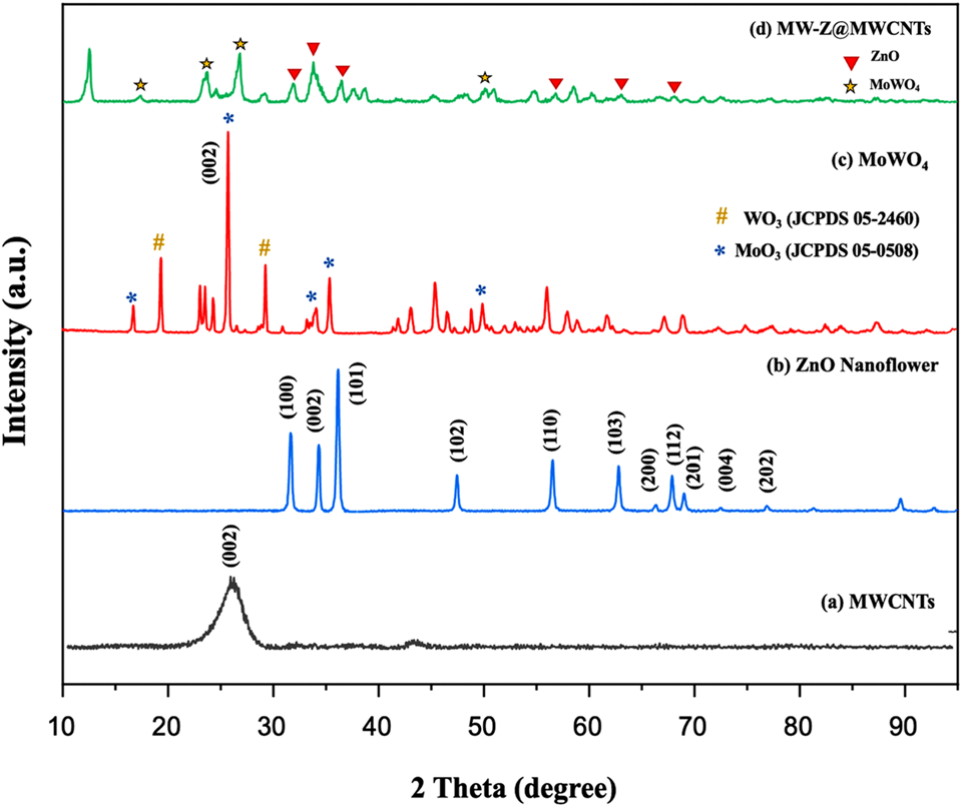
XRD patterns of (**a**) MWCNTs, (**b**) ZnO nanoflowers, (**c**) MoWO_4_, and (**d**) MW-Z@MWCNTs.

The phase structures of the composite samples were determined by XRD (Fig. 2). In the XRD pattern of MWCNTs, a sharp peak appeared at 26.0° corresponding to (002) reflection. The diffraction peaks of ZnO nanoflowers at 2θ = 31.84°, 34.53°, 36.35°, and 49.8° were related to (100), (002), (101), and (102) reflections, confirming that they possessed a hexagonal wurtzite structure (JCPDS 79-0207)^15^. In the XRD spectra of MoWO_4_, the peak appeared from the (002) plane of WO_3_ was stronger than other peaks, and it happened due to the preferred orientation of WO_3_ under the effect of Mo atoms (Fig. 2c). It is confirmed from Fig. 2d that the as-prepared MW-Z@MWCNTs were composed of an interconnected structure.

### Morphological analysis

**Figure 3:**
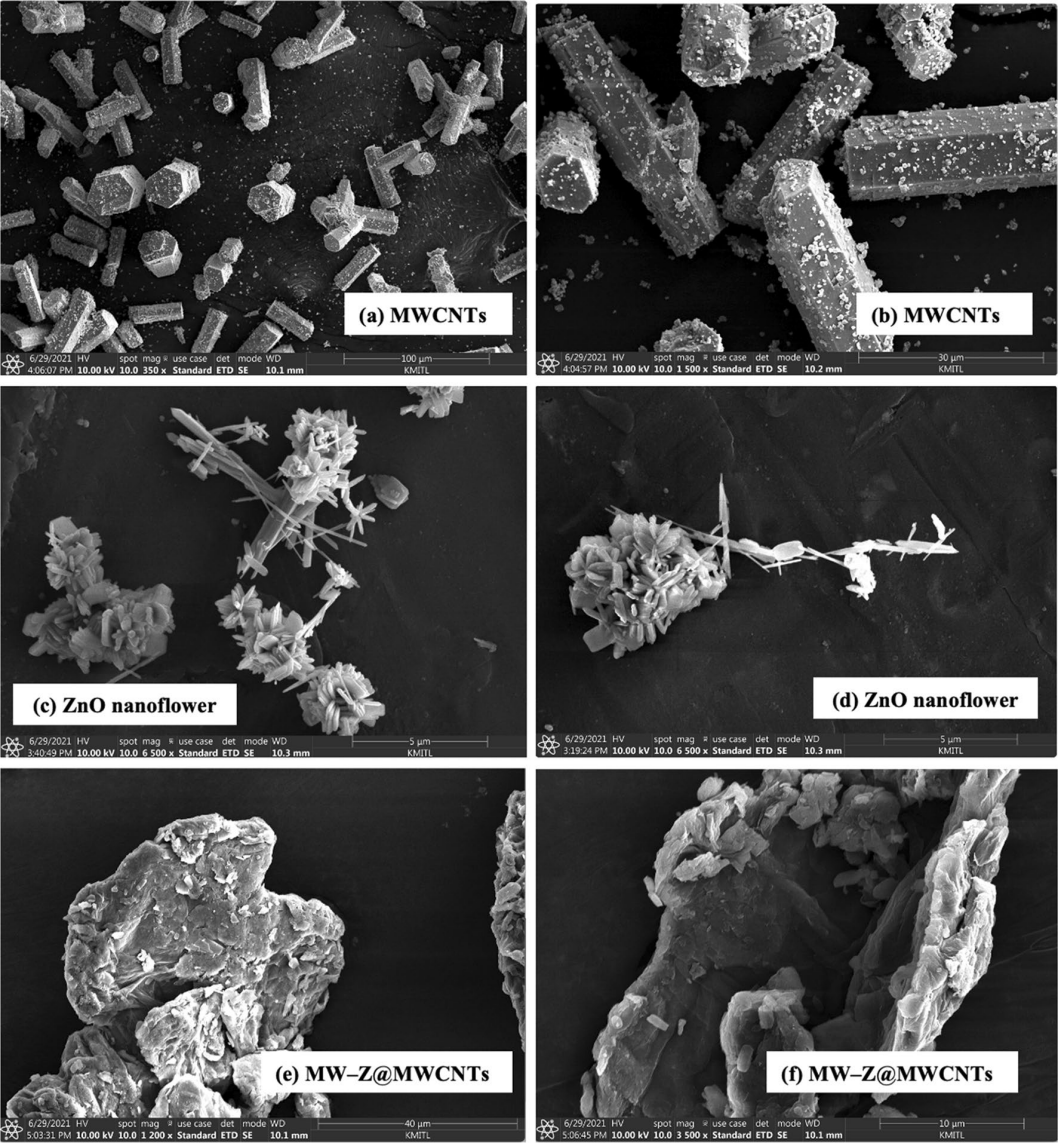
FE-SEM images of (**a,b**) MWCNTs, (**c,d**) ZnO nanoflowers, and (**e,f**) MW-Z@MWCNTs.

**Figure 4:**
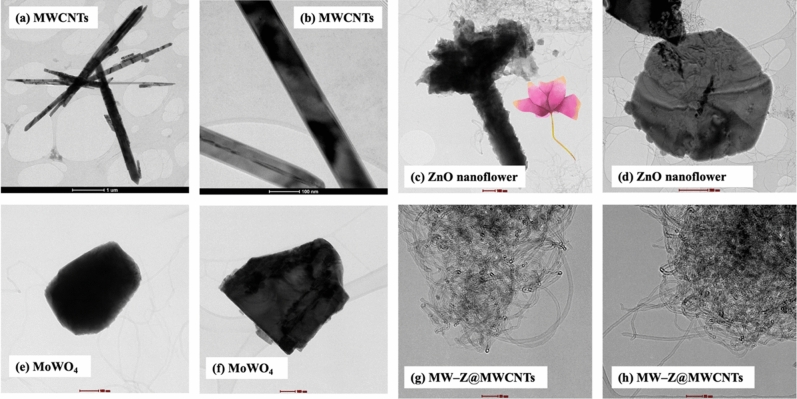
TEM images of (**a,b**) MWCNTs, (**c,d**) ZnO nanoflowers, (**e,f**) MoWO_4_, and (**g,h**) MW-Z@MWCNTs.

The morphology of MW-Z@MWCNTs was identified by FE-SEM and TEM. The morphology and particle size of MWCNTs are displayed in Fig. 3a,b. It is noticeable that MWCNTs consisted of octahedral-shaped overlapping small tubes of 20–30 µm size. It is clear from Fig. 3(c,d) that ZnO nanoflowers contained densely packed nanoneedles. These flower-like clusters were distributed across the surface substrate^16^. The morphology of MW-Z@MWCNTs is presented in Fig. 3(e,f). MW-Z@MWCNTs were found to be clumped together and possessed an indefinite morphology. The internal microstructural arrangement of the as-synthesized samples was determined by TEM (Fig. 4). It is noticeable from Fig. 4a,b that long MWCNTs with 5–50 nm external diameter overlapped each other. Furthermore, the formation of ZnO nanoflowers can be observed in Fig. 4(c,d). In a single nanoflower structure, petal spikes of 600–800 nm size were superficially directed and extended from the center of the flower. In comparison, MoWO_4_ had a relatively flatter morphology with a particle size of about 100 nm. Moreover, the black spots on MW-Z@MWCNTs indicate the attachment of ZnO nanoflowers and MoWO_4_ MWCNTs walls^17^.

### FTIR analysis

**Figure 5:**
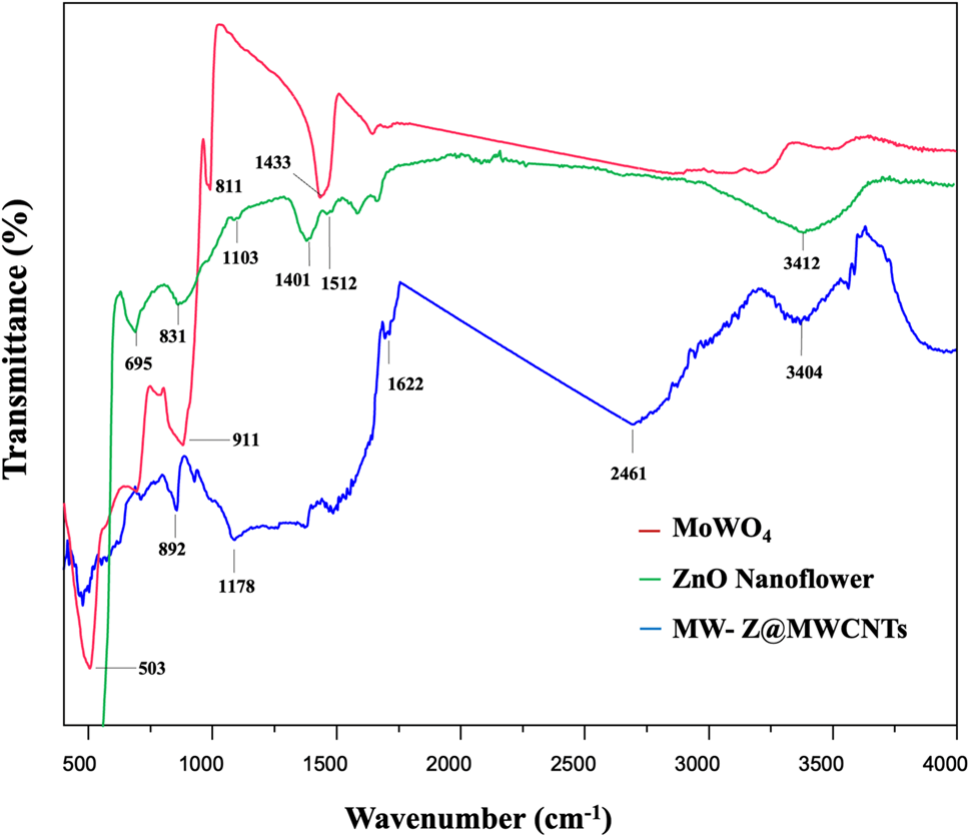
FTIR spectra of MoWO4, ZnO nanoflowers, and MW-Z@MWCNTs.

FTIR spectra in the wavenumber range of 400–4000 cm^−1^ were used to determine the chemical bonding state of ZnO nanoflowers, MoWO_4_, and MW-Z@MWCNTs (Fig. 5). In the spectrum of MoWO_4_, the bands at 811 cm^−1^ and 503 cm^−1^ appeared from the symmetrical vibration of Mo–W–O groups and the stretching vibration of Mo–O bonds, respectively. Moreover, the strong band at 620 cm^−1^ was formed due to the asymmetrical stretching vibration of W–O bonds in (W_2_O_4_)_n_ chains^18^. In the spectrum of ZnO nanoflower, the characteristic band at 3412 cm^−1^ appeared from the stretching vibration of hydroxyl (OH) groups. The small peak around 1103 cm^−1^ could be assigned to the symmetric stretching of C–O–C bonds stretching mode. The deformation of OH groups occurred at 1401 cm^−1^. The peak around 1512 cm^−1^ could be attributed to the strong mode of vibration of C=O^19^. Moreover, the peaks around 695 cm^−1^ and 879 cm^−1^ appeared from the hexagonal phase of ZnO and the stretching vibration of C–O, respectively. In the spectrum of MW-Z@MWCNTs, the absorption bands at 3404 cm^−1^ and 1622 cm^−1^ appeared due to O–H bonding on the surface of MWCNTs. The peak at 892 cm^−1^ indicates the presence of O-C bonds in purified MWCNTs^20^.

### Raman spectroscopy

**Figure 6:**
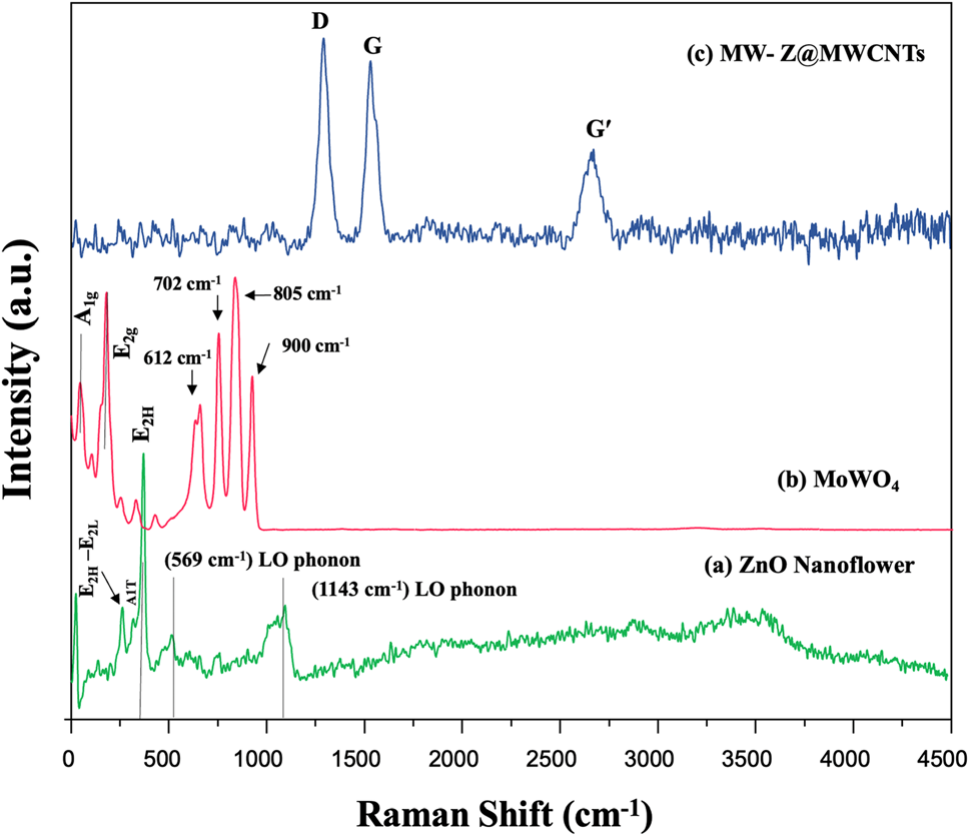
Raman spectra of (**a**) ZnO nanoflowers, (**b**) MoWO_4_, and (**c**) MW-Z@MWCNTs.

Raman spectroscopy was used to characterize intrinsic defects in ZnO nanoflowers, MoWO_4_, and MW-Z@MWCNTs (Fig. 6). The E_2H_–E_2L_ and A_1T_ modes at ∼331 and ∼380 cm^−1^, respectively, appeared from ZnO nanoflower crystals (line (a)). The peaks at ∼436 and ∼583 cm^−1^ could be assigned to the E_2H_ and E_1L_ phonon modes, respectively. The E_2H_ mode represents a typical wurtzite crystal structure and reflects a perfect ZnO nanoflower crystal property. In addition, the centered peak of ZnO nanoflower at 569 cm^‒1^ and 1143 cm^‒1^ which corresponded to the first, second and third-order LO phonon bands of ZnO. To further obtain more information about structural and presence or absence of vibrational modes Raman spectra for MoWO_4_ shown in Fig. 6b, The Raman bands at 109 cm^−1^ and 225 cm^−1^ appeared from the A_1g_ and E_2g_ phonon modes and were related to the layered 2H phase of MoWO_4._ The Raman bands at 612, 702, 805 and 900 cm^‒1^ have been linked to the υ(O–W–O) stretching vibrational modes. In the Raman spectrum of MW-Z@MWCNTs, the peak at 1306 cm^−1^ corresponds to the defect (D) band (disorder-induced structures, tube ends, staging disorder), the peak at 1502 cm^−1^ corresponds to crystalline graphitic structures (G band), and the peak at 2728 cm^−1^ corresponds to the replica of the D band^21,22^.

### XPS analysis

**Figure 7:**
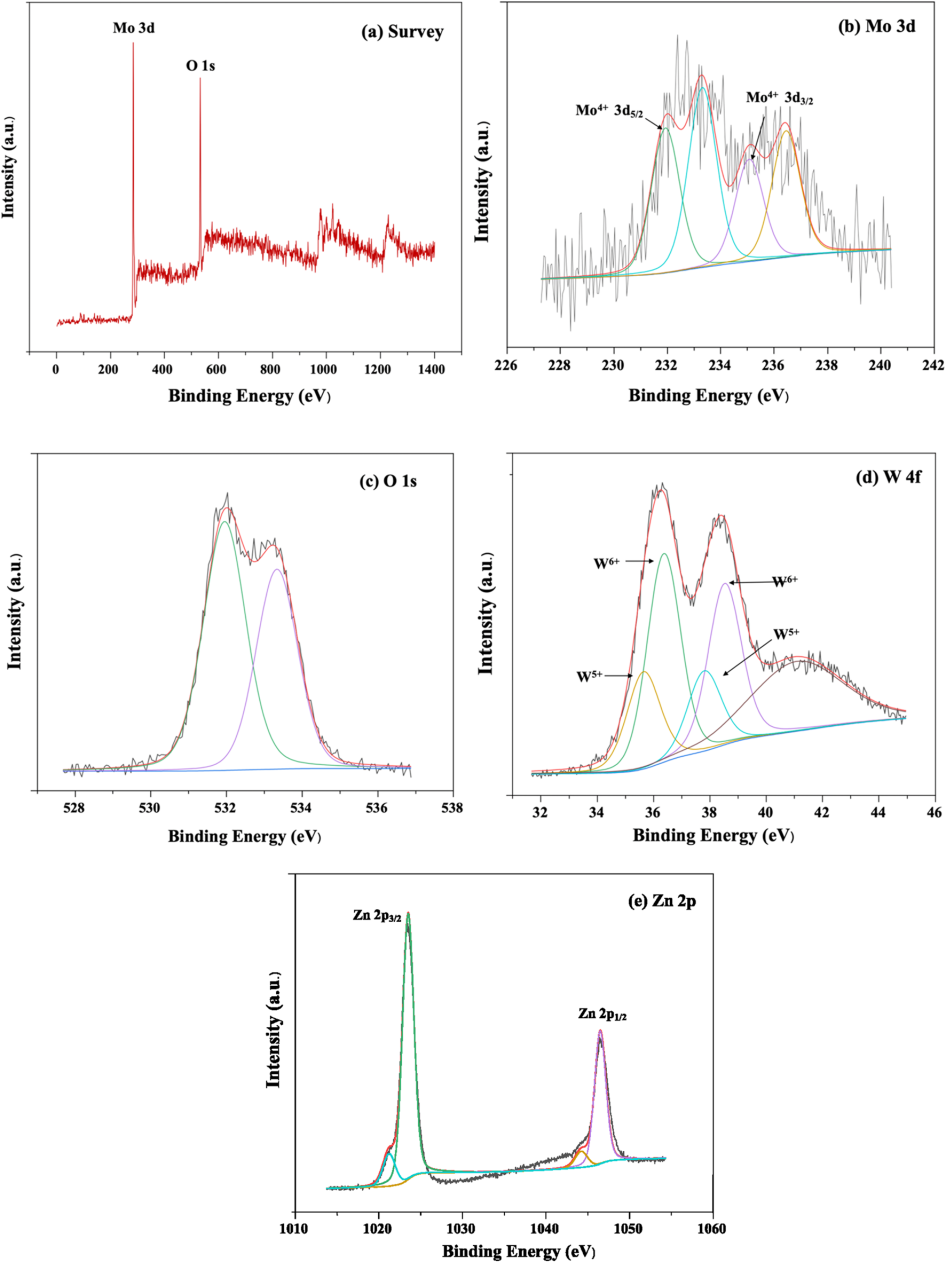
XPS spectra of (**a**) survey, (**b**) Mo 3d, (**c**) O1s, (**d**) W 4f, and (**e**) Zn 2p.

The XPS spectra of MW-Z@MWCNTs are displayed in Fig. 7. The presence of Mo, W, Zn, and O was well detected in MW-Z@MWCNTs (Fig. 7a). Figure 7b presents the high-resolution XPS spectrum of the Mo 3d peak. The binding energies of Mo 3d_3/2_ and Mo 3d_5/2_ were calculated as 235.0 eV and 231.5 eV, respectively, indicating the existence of Mo atoms in the + 4 oxidation state^23^. Moreover, the peaks at 531.4 eV and 533.1 eV could be assigned to oxygen atoms in MoWO_4_ (Fig. 7c). The spin–or-bit doublets at 35.6 eV, 37.6 eV, and 41.5 eV in the W 4f spectrum correspond to the W 4f_7/2_, W 4f_5/2_, and W 5p_3/2_ peaks, respectively, indicating the existence of W atoms in the + 6 oxidization state^24^. In Fig. 7e, the binding energies of 1021.70 eV and 1045.78 eV could be assigned to the Zn 2p_3/2_ and Zn 2p_1/2_ peaks, respectively.

### N_2_ adsorption–desorption isotherm (BET) analysis

**Figure 8:**
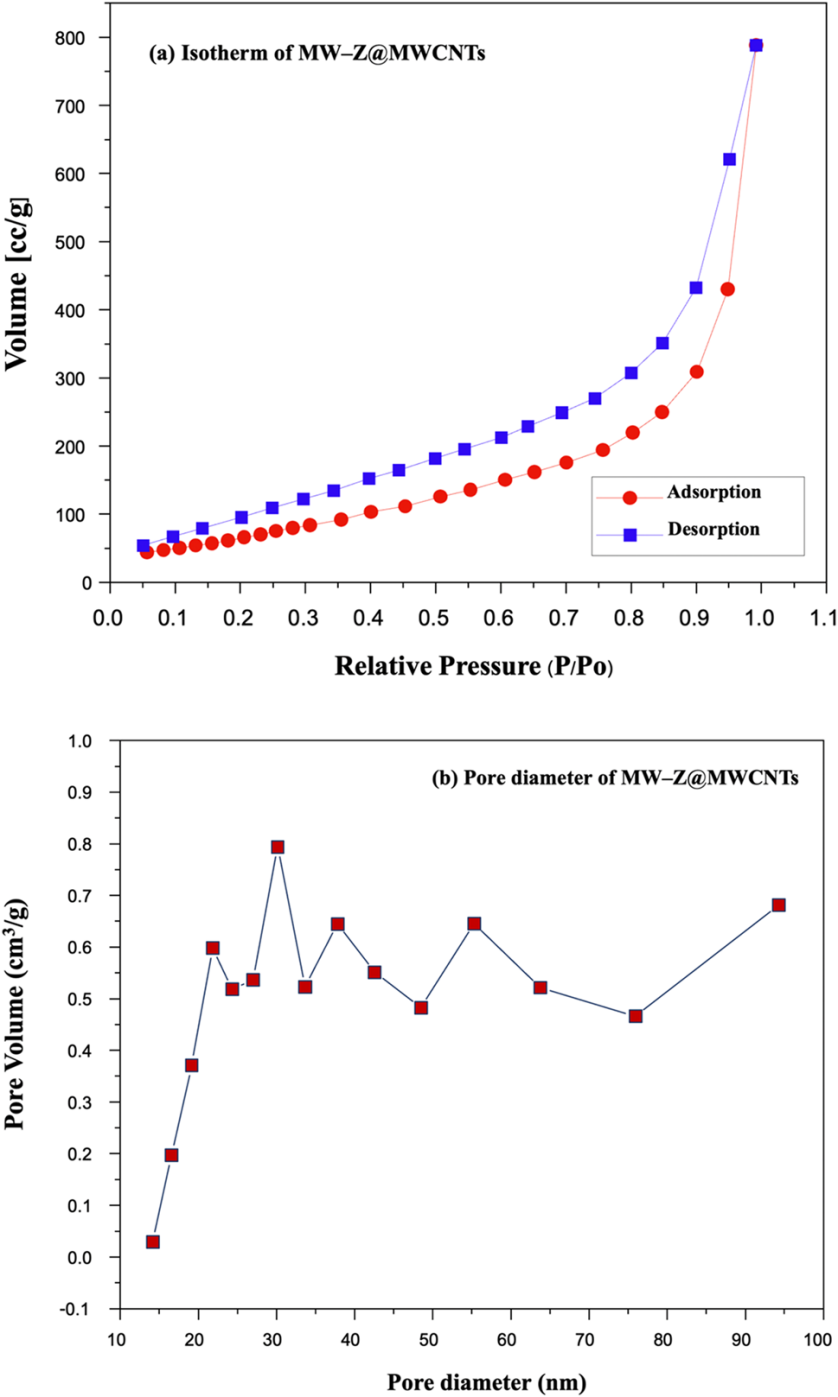
(**a**) Typical N_2_ adsorption–desorption isotherm and (**b**) pore size distribution curve of MW-Z@MWCNTs.

N_2_ adsorption–desorption measurements were performed to measure the specific surface areas of MW-Z@MWCNTs (Fig. 8). In a typical BET analysis, a measure of the specific surface area (SSA) of MW-Z@MWCNTs is determined from the volume of N_2_ gas adsorbed on the MW-Z@MWCNTs. The basics Brunauer, Emmett, and Teller (BET) theory, the most common method used to describe the specific surface area followed the equation:

1 $$1/{\text{W}}\left( {\left( {{\text{P}}_{0} /{\text{P}}} \right) - {1}} \right)\, = \,\left( {{1}/{\text{W}}_{{\text{m}}} {\text{C}}} \right)\, + \,\left( {{\text{C}} - {1}/{\text{W}}_{{\text{m}}} {\text{C}}} \right)\left( {{\text{P}}/{\text{P}}_{0} } \right)$$

where *W* is the weight of gas adsorbed, *P/P*_*0*_ is the relative pressure, *Wm* is the weight of adsorbate as a monolayer, and C is the BET constant. Type II isothermals with H_3_ hysteresis could be attributed to the mesoporous structure of the samples^25^. Moreover, the large specific surface area (30.33 m^2^g^–1^) and pore size (about 30 nm) of MW-Z@MWCNTs significantly facilitated the access of the electrolyte and allowed rapid charge transfer kinetics to improve the power conversion efficiency of DSSC.

### Photovoltaic study of DSSCs with different counter electrodes

**Figure 9:**
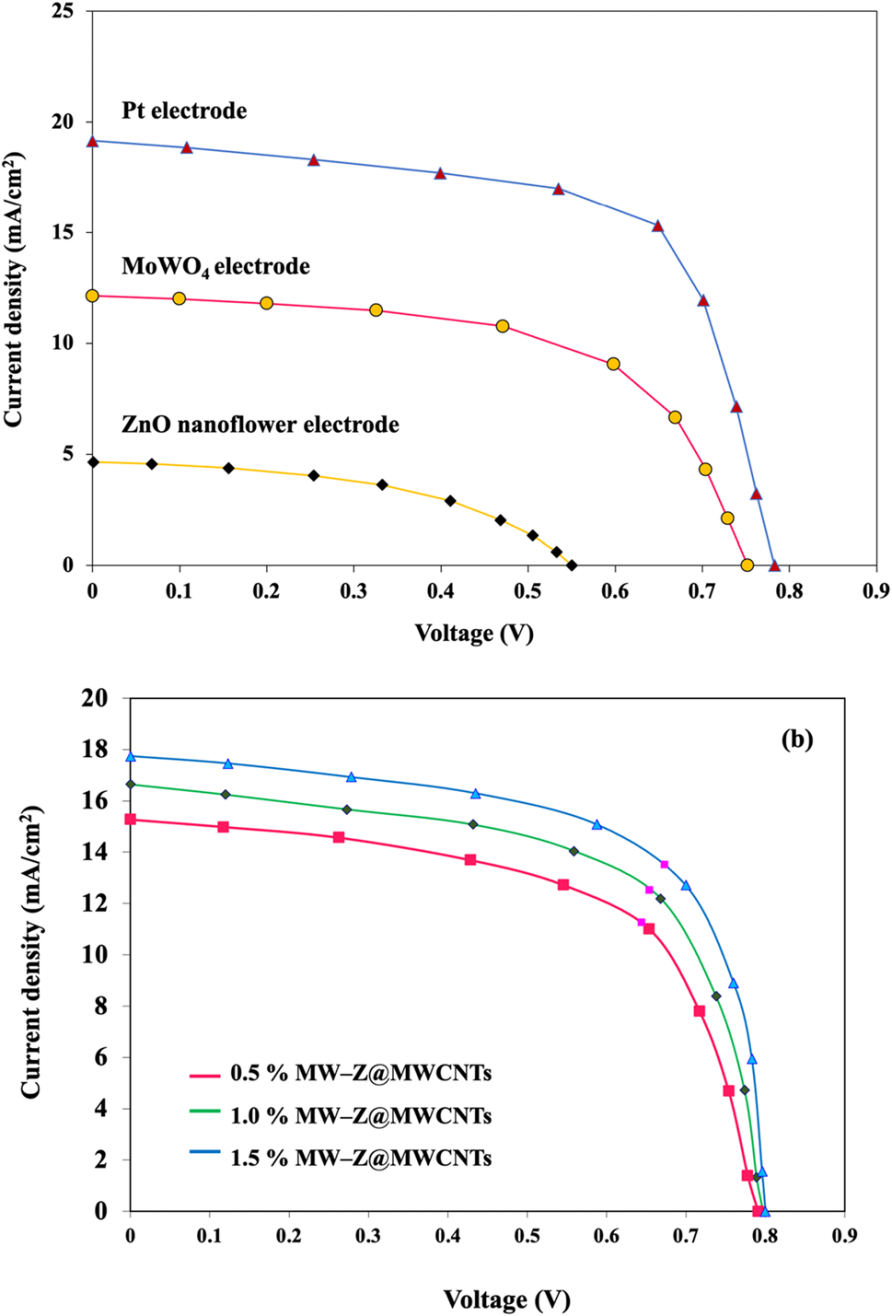
Current–voltage characteristics of DSSCs with different Ces.

The PCE values of different CEs for DSSC were calculated by a Class AAA solar simulator under AM 1.5G simulated sunlight (Fig. 9 and Table 1). The 1.5% MW-Z@MWCNTs CE yielded the highest power conversion efficiency of 9.96% with Voc = 0.78 V, Jsc = 19.16 mA/cm^2^, and FF = 0.66 followed by 1.0% MW-Z@MWCNTs (PCE = 8.19%) and 0.5% MW-Z@MWCNTs (PCE = 7.25%), which are greatly higher than those of traditional Pt CE, pure ZnO nanoflower CE, and MoWO_4_ CE under the same conditions. The higher PCE of MW-Z@MWCNTs could be attributed to the presence of ZnO nanoflowers and MoWO_4_ on MWCNTs, resulting in a large surface area and good charge electron transport capacity, which synergistically improved the electrocatalytic activity of the CE. In addition, the higher Voc of 1.5% MW-Z@MWCNTs could be ascribed to the efficient regeneration of the N719 dye with this CE, resulting in the generation of a greater number of excitons. The high surface area for adsorption capacity, the MWCNTs, and ZnO nanoflower result in an improvement in electron–hole pair production. Moreover, the enhanced performance is due to an improved conductive path between the MoWO_4_ nanoparticles by the addition of MWCNTs which displays remarkable catalytic activity in the reduction of I^−^_3_^26^.

**Table 1:** Photovoltaic parameters of DSSCs with different CEs.

Sample	Voc (V)	Jsc (A/cm^2^)	FF	PCE (%)	Rs (Ω)	Rct (Ω)
Pt electrode	0.12	13.51	0.64	9.09	10..56	14.78
ZnO nanoflowers	0.55	4.64	0.48	1.22	10.15	40.21
MoWO_4_	0.72	12.14	0.59	5.44	10.05	31.12
0.5% MW-Z@MWCNTs	0.79	11.27	0.60	7.25	10.22	35.2
1.0% MW-Z@MWCNTs	0.79	12.53	0.61	8.19	10.12	15.8
1.5% MW-Z@MWCNTs	0.78	19.16	0.66	9.96	10.01	7.51

### Electrochemical impedance spectroscopy (EIS) analysis

**Figure 10:**
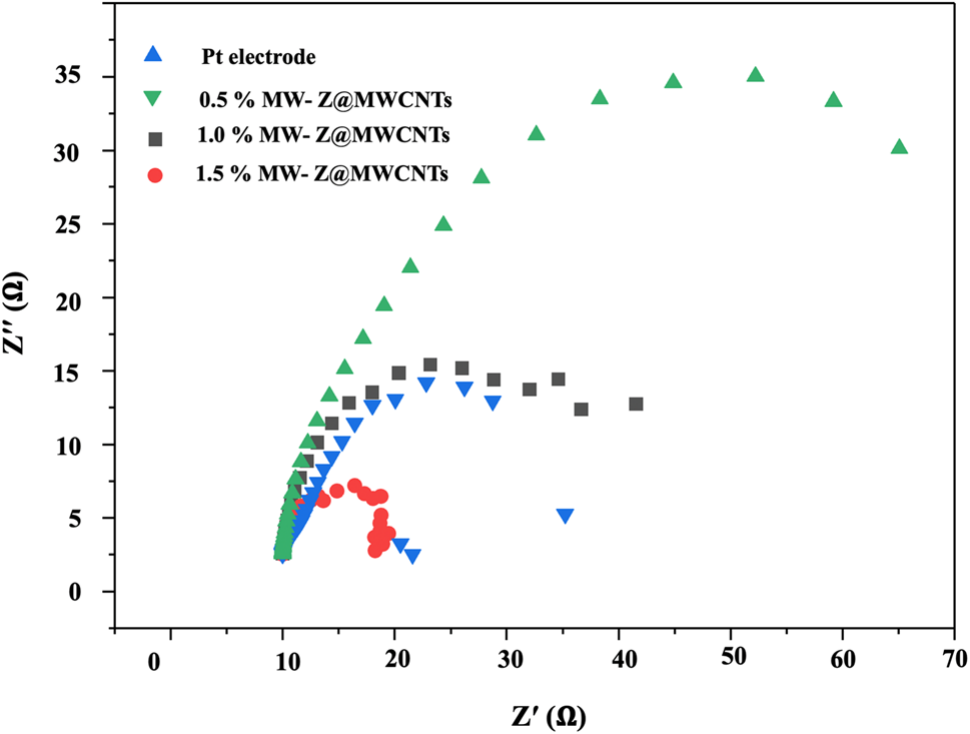
EIS analysis of the fabricated DSSCs with ZnO nanoflowers, MoWO_4_, and MW-Z@MWCNTs.

The Nyquist plots of different CEs were measured to determine the ionic and electronic transport process of the DSSCs as shown in Fig. 10 and Table 1. Normally, two apparent semi-circles are detected in the Nyquist plots. The small arc at a high frequency is attributed to the resistance between the counter electrode and electrolyte mediator. The large arc at the mean frequency is associated with charge transfer resistance (R_ct_) at the interfaces of MW-Z@MWCNTs with electrolyte and dye molecules. The resistance element R_S_ in the high-frequency region (> 10^5^ Hz) is ascribed to the sheet resistance of the FTO substrates. Similar resistance for counter electrode (R_CE_), deduced from high-frequency semi-circle, implies that we used the Pt as a reference counter electrode by replacement of MW-Z@MWCNTs through our experiments. The R_ct_ value of the 1.5% MW-Z@MWCNTs CE was lower than those of other electrodes. The R_ct_ value increased greatly with the decrease of MWCNTs percentage because the addition of MWCNTs significantly improved the charge transfer capability of MW-Z@MWCNTs CE. Furthermore, the 1.5% MW-Z@MWCNTs CE enhanced the electrocatalytic effect of accessible and interconnected pores in ZnO nanoflowers and MWCNTs, allowing more efficient electron transfer between the electrode and the electrolyte to enhance the reduction reaction of the redox couple.

## Conclusion

Novel MW-Z@MWCNTs were successfully synthesized by a simple hydrothermal route for CE applications in DSSC. The chemical states, morphologies, and catalytic properties of ZnO nanoflowers, MoWO_4_, and MW-Z@MWCNTs were analyzed. The 1.5% MW-Z@MWCNTs structure exhibited better I^−^_3_ electrocatalytic activity than Pt CE under the same conditions. The addition of MWCNTs greatly enhanced the electron charge transport capacity and surface area of ZnO nanoflowers. Hence, this unique MW-Z@MWCNTs structure can serve as an efficient CE for Pt-free DSSCs.

## Data Availability

The data that support the findings of this study are presented in the main text and are available from the corresponding author upon request.
